# Optimization of a nicotine degrading enzyme for potential use in treatment of nicotine addiction

**DOI:** 10.1186/s12896-019-0551-5

**Published:** 2019-08-02

**Authors:** Thomas Thisted, Zuzana Biesova, Celine Walmacq, Everett Stone, Max Rodnick-Smith, Shaheda S. Ahmed, Stephen K. Horrigan, Bo Van Engelen, Charles Reed, Matthew W. Kalnik

**Affiliations:** 1 Antidote Therapeutics, Inc, 708 Quince Orchard Road, Suite 250-C, Gaithersburg, MD 20878 USA; 2 Department of Molecular Biosciences, The University of Texas at Austin, Austin, TX USA; 3 Alcyomics Ltd, Bulman House, Regent Centre, Gosforth, Newcastle upon Tyne, NE3 3LS UK; 4 Noble Life Sciences, PO Box 242, Woodbine, MD 21797 USA; 5 Maastricht University, P. Debyeplein 1, 6229 HA, Maastricht, NL USA

**Keywords:** Nicotine, Enzyme, Metabolism, Degradation, Addiction, Smoking cessation, Protein engineering, Enzyme catalysis, Nicotine-degrading enzyme, Drug design

## Abstract

**Background:**

Smoking and tobacco use continue to be the largest preventable causes of death globally. A novel therapeutic approach has recently been proposed: administration of an enzyme that degrades nicotine, the main addictive component of tobacco, minimizing brain exposure and reducing its reinforcing effects. Pre-clinical proof of concept has been previously established through dosing the amine oxidase NicA2 from *Pseudomonas putida* in rat nicotine self-administration models of addiction.

**Results:**

This paper describes efforts towards optimizing NicA2 for potential therapeutic use: enhancing potency, improving its pharmacokinetic profile, and attenuating immunogenicity. Libraries randomizing residues located in all 22 active site positions of NicA2 were screened. 58 single mutations with 2- to 19-fold enhanced catalytic activity compared to wt at 10 μM nicotine were identified. A novel nicotine biosensor assay allowed efficient screening of the many primary hits for activity at nicotine concentrations typically found in smokers. 10 mutants with improved activity in rat serum at or below 250 nM were identified. These catalytic improvements translated to increased potency in vivo in the form of further lowering of nicotine blood levels and nicotine accumulation in the brains of Sprague-Dawley rats. Examination of the X-ray crystal structure suggests that these mutants may accelerate the rate limiting re-oxidation of the flavin adenine dinucleotide cofactor by enhancing molecular oxygen’s access. PEGylation of NicA2 led to prolonged serum half-life and lowered immunogenicity observed in a human HLA DR4 transgenic mouse model, without impacting nicotine degrading activity.

**Conclusions:**

Systematic mutational analysis of the active site of the nicotine-degrading enzyme NicA2 has yielded 10 variants that increase the catalytic activity and its effects on nicotine distribution in vivo at nicotine plasma concentrations found in smokers. In addition, PEGylation substantially increases circulating half-life and reduces the enzyme’s immunogenic potential. Taken together, these results provide a viable path towards generation of a drug candidate suitable for human therapeutic use in treating nicotine addiction.

## Background

Smoking is a global healthcare problem [1]. The World Health Organization estimates that there are 1.1 billion smokers worldwide today and over 7 million tobacco-related deaths each year [2]. According to the United States Center for Disease Control, tobacco use is the single leading preventable cause of death in the U.S., responsible for approximately 438,000 deaths each year [3]. Currently available pharmacotherapies for the treatment of tobacco use disorder (nicotine replacement therapy, varenicline and bupropion) have been helpful for enhancing smoking cessation rates, but most quit attempts still end in failure [4, 5]. Thus, a major need exists for a more effective approach to achieving long-term smoking abstinence.

Recently, a biotherapeutic strategy has been proposed: degradation of nicotine in blood via an enzymatic approach in order to reduce nicotine delivery to the brain [6]. Pre-clinical proof of concept has been obtained dosing the amine oxidase NicA2 from *Pseudomonas putida* in well-accepted rat behavioral models of nicotine addiction [7, 8].

*Pseudomonas putida S16* is an example of a nicotine-degrading bacterium that can use nicotine as its sole nitrogen and carbon source. It was originally isolated from a field under continuous tobacco cropping in China and is able to metabolise nicotine to fumaric acid [9]. The enzyme catalyzing the first committed step of S16’s degradation of nicotine is NicA2. NicA2 oxidizes nicotine into N-methylmyosmine which undergoes rapid, spontaneous hydrolysis to pseudooxynicotine (PON) [6], a non-addictive, nicotine metabolite also produced endogenously in smokers [10]. Initial in vitro studies of NicA2 demonstrated favourable characteristics such as high stability in buffer as well as mouse serum, and good catalytic activity at nicotine concentrations typically found in smoker’s blood [6]. In addition, 5-day and 5-week toxicity studies of PON, intravenously (*i.v.*) administered daily or every other day, respectively, showed no evidence of health or behavioral problems over a range of doses in mice [6].

NicA2 has been evaluated in vivo in single-dose nicotine pharmacokinetic (PK) studies in rats pre-treated with a range of NicA2 doses [7, 11]. Reduction in nicotine blood and brain levels were measured 1, 3, and 5 minutes (min) after an *i.v.* bolus dose of 0.03 mg/kg nicotine administered in < 10 s via jugular venous catheter which provides an initial spike in nicotine concentration in both blood and brain. The administered nicotine dose in rats was equivalent to the nicotine absorbed from smoking 2 cigarettes, based on the amount of nicotine in milligrams per kilogram of body weight. When NicA2 was dosed at 5 mg/kg, blood levels of nicotine dropped to below the limit of quantitation (LOQ) of the assay (2 ng/mL), virtually eliminating nicotine from the bloodstream within 1 min, as compared to control rats receiving bovine serum albumin. The levels of nicotine in the brain were also assessed and a 20 mg/kg NicA2 dose lowered brain nicotine levels by 95% at 1 min after nicotine dosing, as compared to controls. These results are important since the reinforcing effects of nicotine are greatest in the first few minutes after a nicotine dose and the enzyme’s clinical effectiveness is expected to be dependent upon rapid elimination of nicotine.

A NicA2-albumin binding domain (ABD) fusion with extended circulating half-life was shown to attenuate nicotine discrimination and to decrease nicotine reinforcement in a continuous nicotine access self-administration model, which closely resembles human nicotine exposure [7]. After a high dose of NicA2-ABD (70 mg/kg), nicotine seeking behaviour was extinguished over 6 days (d) of testing with rats having continuous access to nicotine [7]. Furthermore, chronic administration of NicA2 reversed signs of withdrawal, hyperalgesia, and irritability-like behavior in nicotine-dependent rats, as well as prevented nicotine- and stress-induced relapse [8]. The observation that NicA2 does not induce withdrawal symptoms is important as the negative affective consequences of early nicotine withdrawal are recognized as significant contributors to relapse to tobacco smoking during quit attempts [12–14], and the maintenance of compulsive nicotine use [15–17]. In addition, the enhancement by nicotine of the reward value of other environmental rewarding stimuli is considered critical in the maintenance of nicotine dependence [18–24]. Thus, blockade of nicotine-induced reward enhancement without inducing strong withdrawal effects are desirable properties of NicA2. This putative anti-smoking medication may play an important role in preventing relapse within the quit process and in maintening abstinence. These initial studies provided proof of concept for the use of NicA2 as a basis for development of a smoking cessation drug [7, 8].

Kinetic characterization of NicA2 indicated a low *K*_m_ (92 nM at 37 °C which compares favorably to the serum nicotine concentration in a typical smoker of 250 nM), but a relatively slow catalytic turnover rate [6]. This could be part of the reason for the relatively high doses required for efficacy in the rat nicotine-self-administration studies [7]. While mg/kg dose levels in this animal model do not necessarily reflect human dose levels (e.g. efficacious varenicline dose was found in this model to be 50-fold higher than typically used in the clinic [25]), improvements in catalytic activity will be important for achieving lower dose levels, as well as suitable routes and frequency of drug administration.

While the structure of NicA2 has been elucidated [26, 27], very limited knowledge about the role and importance of individual residues in catalytic activity exists, and to date only 2 rationally designed mutations, T381V and T250V/T381V, have been reported in the literature; each showing a decrease in catalytic efficiency (*k*_cat_/*K*_m_) in vitro [27]. We have taken the approach of screening libraries randomizing all active site positions to start collecting empirical data on the effect on enzymatic activity by mutating various residues. A comprehensive data set of 58 single point mutations with enhanced activity at 10 μM substrate concentration compared to wild-type (wt) was obtained, and a small subset of these (10) were shown to have enhanced activity in serum at nicotine concentrations relevant in smokers. In vivo data comparing wt and the newly discovered variant A107R suggest the in vitro improvement translates to enhanced in vivo potency. Furthermore, we present data showing random PEGylation of NicA2 prolongs its circulating half-life while at the same time diminishing the immunogenic potential of this bacterial enzyme, without impacting its catalytic activity.

## Results

### High-throughput primary screening assay developed with parallel affinity purification and enzymatic activity assessment

**Fig. 1:**
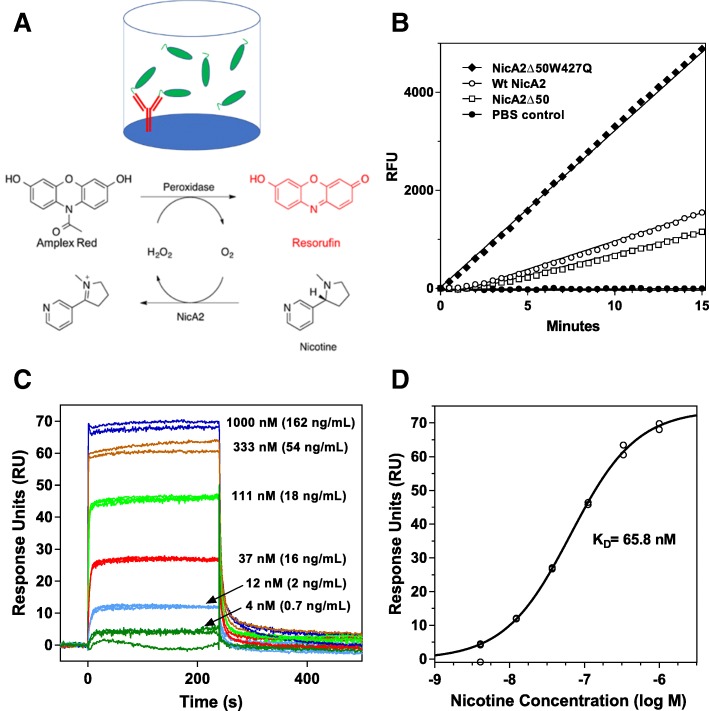
Primary and secondary enzyme screening assays**.** Primary 96-well plate assay (10 μM S-(−)-nicotine): (**a**) The activity of a fixed amount of NicA2-His (green) captured by anti-His mAb (red) immobilized in each assay well is monitored by the Amplex Red assay measuring the formation of the fluorescent product Resorufin over time, (**b**) An activity assay using purified proteins comparing wt NicA2 (open circle) to NicA2Δ50 (open square) and NicA2Δ50W427Q (solid diamond). The relative slopes of the curves of Relative Fluorescence Units (RFU) vs. time were 1, 0.7 and 3.6, respectively. Secondary biosensor assay (250 nM S-(−)-nicotine): (**c**) Sensorgram of 3-fold serial dilution of S-(−)-nicotine in running buffer passed over a sensor surface with immobilized anti-nicotine antibody over a range of nicotine concentrations (as indicated, in triplicate). After a steady-state level of binding was achieved, running buffer without nicotine was passed over the chip (starting at t = 240 s) to dissociate nicotine from the mAb. (**d**) Plot of sensor Response Units (RU) at steady-state (from experiment in panel C) vs. S-(−)-nicotine concentration

A high-throughput screening assay was implemented, in which NicA2 variants could be expressed, purified and assayed for specific activity in parallel in a 96-well plate format. As illustrated in Fig. 1a, NicA2 activity was measured in a coupled Amplex Red enzyme assay [28]. His-tagged variants were expressed in parallel in 96-well deep-well plates, and affinity purified from the crude *E. coli* lysates in each assay-well on immobilized anti-His monoclonal antibody (mAb) (Fig. 1a). Capturing a very small fraction of the total expressed NicA2-His present in the cell lysates, by a constant amount of capture antibody, results in a normalization of the amount of enzyme in the assay, and the activities measured are consequently not influenced by variations in growth, induction of expression and intrinsic expression levels. The assay displays very little well-to-well variation, and a 2-fold activity increase was chosen as a cut-off for hit identification (see Methods).

In an effort to eliminate any non-essential bacterial sequence (including a specific in silico predicted T-cell epitope sequence [29]) to reduce immunogenic risk, a deletion construct was made removing the first 50 N-terminal residues which appeared unstructured in the crystal structure [26], resulting in the construct NicA2Δ50. Figure 1b shows the Amplex Red assay results (shown as calculated slopes of Relative Fluorescence Units (RFU) as a function of time) for purified NicA2 and NicA2Δ50 enzymes. Purified NicA2Δ50 displayed a marginal 23% reduction in activity relative to full-length wild-type NicA2 (Fig. 1b and Table 1).

**Table 1:** Primary enzyme assay hits

Position	NicA2 variant	Fold improvement over wt
	wt	1
	Δ50	0.7
91	Δ50R91A	3.8
	R91Q	3.3
	Δ50R91Q	3.1
	Δ50R91F	2.7
	Δ50R91G	2.5
	Δ50R91T	2.4
	Δ50R91L	2.4
	Δ50R91S	2.1
104	F104R	6.4
	F104K	5.9
	F104I	3.8
	F104 L	3.5
	F104S	3.2
	F104 T	2.3
106	G106S	3.9
	G106A	3.8
107	A107R	19.0
	Δ50A107R	17.3
	A107K	6.7
	A107T	5.9
	A107G	4.9
	A107H	4.8
	A107P	3.5
217	Δ50L217Q	2.8
	Δ50L217G	2.3
	Δ50L217E	2.0
249	E249W	5.5
	E249D	2.7
250	T250G	4.7
	Δ50T250L	3.5
340	Δ50K340P	2.1
355	F355H	13.4
	F355K	4.6
	F355C	2.6
366	Δ50Q366K	4.9
	Δ50Q366E	3.2
	Q366K	3.1
	Δ50Q366V	2.4
	Δ50Q366L	2.2
381	Δ50T381P	5.5
	Δ50T381I	5.4
	Δ50T381V	4.9
	Δ50T381Q	4.3
	Δ50T381N	2.7
	Δ50T381L	2.4
	Δ50T381M	2.1
426	A426Q	5.2
	A426W	4.9
427	Δ50W427S	3.3
	Δ50W427E	3.1
	Δ50W427Q	3.0
	Δ50W427M	2.8
462	Δ50N462L	6.5
	Δ50N462Y	5.7
	Δ50N462S	4.2
	N462Y	3.2
	Δ50N462F	3.1
	Δ50N462G	3.0
	Δ50N462E	2.6
	Δ50N462A	2.2
463	Δ50I463F	3.7
	Δ50I463Y	3.5

The 58 single amino acid variants identified at 15 unique positions within the set of 22 active site residues (listed in Experimental Procedures) with enzymatic activity at least 2-fold greater than wt NicA2 in the Amplex Red primary screening assay

To validate the primary screening assay, we compared NicA2Δ50 to the very first screening hit we isolated, NicA2Δ50 with the W427Q point mutation. In the primary screening assay, the W427Q mutation increased activity 3-fold over wt (Table 1). Figure 1b shows the Amplex Red assay results comparing fully purified and quantitated NicA2Δ50 and NicA2Δ50W427Q enzymes. The 3-fold enhanced activity of the W427Q mutation detected in the primary screening assay in lysate translated to a 3.6-fold increased activity for the fully purified variant compared to wt.

In order to test whether mutations improving activity in the NicA2Δ50 backbone caused similar improvements in the full-length enzyme, 6 mutations (R91Q, A107R, Q366K, T381V, W427Q and N462Y identified as described below) were tested in both backbones. All tested mutations provided similar activity enhancements in both the NicA2Δ50 backbone as well as the full-length enzyme, with full correlation between primary screening assay format and the secondary assay using purified enzyme.

### Primary screening of active-site variant libraries identified 58 novel point mutations with increased catalytic activity

A set of 22 active site residues were identified based on the published crystal structure (described in Methods). Individual saturation mutagenesis libraries were generated based on NNK randomization (i.e. using an oligonucleotide containing a mixture of all 4 bases in the first two positions and a G or T for the third position encoding all 20 amino acids in the codon of interest) for 8 positions for initial screening, method development and validation. To increase laboratory productivity, the remaining 13 positions were concurrently incorporated into a single custom Comprehensive Saturation Mutagenesis (CSM) library with each variant carrying a single NNK-encoded point mutation [30]. Two 96-well plates were screened from each of the 8 individual NNK libraries, while a total of 12 plates were screened from the CSM library (see Methods for details). DNA sequencing was carried out to identify the mutations in each confirmed screening hit. The average activities for each variant reported relative to wt are listed in Table 1. A total of 58 individual hits were obtained at 15 unique positions within the 22 active site residues.

### Secondary screening determined 10 of the 58 novel mutants maintained improved catalytic activity at the low serum nicotine levels found in smokers

**Fig. 2:**
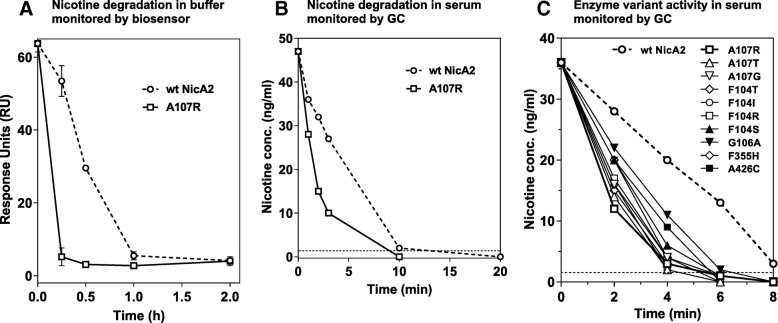
Secondary enzyme screening assays at nicotine concentrations found in smokers: Activity of purified enzyme variants. (**a**) His-tagged wt NicA2 enzyme (circles) or mutant NicA2A107R (squares) were captured from *E. coli* lysates by anti-His-tag mAb in assay wells. Buffer containing 250 nM S-nicotine was added, samples retrieved, and heat inactivated at the time points indicated. Samples were run on the biosensor instrument, and Response Units were plotted as a function of time. (**b**) Purified enzyme or mutant NicA2A107R were added to rat serum containing 40 ng/mL S-(−)-nicotine, and samples were withdrawn and activity quenched by MeOH addition at the time points indicated. Residual nicotine concentration was measured by GC. (**c**) Enhanced activity of 10 variants over wt NicA2 at 40 ng/mL nicotine in serum. Each purified variant (1.5 μg/mL) was added to rat serum containing 40 ng/mL nicotine at 37 °C, and activity quenched with methanol at various timepoints. Open symbols indicate increased activity compared to wt of > 3-fold in this assay. Nicotine concentrations were measured by GC

With a total of 58 individual variants with enhanced activity at 10 μM substrate (a concentration 100-fold over the published *K*_m_ for wt NicA2 [6]; reflecting an enhanced *k*_cat_ over wt), a novel secondary screening assay was designed to efficiently identify those variants that also had improved activity at the lower nicotine concentrations encountered in the blood of a smoker (250 nM; typical daily maximum) [31]. We implemented an automated nicotine biosensor assay, that enabled efficient and accurate screening of variants without requiring full purification and quantification. As shown in Fig. 1c and d, using Surface Plasmon Resonance (SPR), a nicotine-specific mAb immobilized on a sensor chip can give response curves where the SPR signal (expressed in response units, RU) at steady-state is proportional to the nicotine concentration in the buffer passed over the chip surface. This assay allowed for quantitation of unknown nicotine levels in buffer in a range of single digit nM to 1 μM for this particular antibody (ATI-1119) with a dissociation constant (*K*_D_) for S-(−)-nicotine of 66 nM. We devised an assay setup where residual nicotine concentrations could be measured as a function of incubation time with enzyme. As seen in Fig. 2a, a decrease in RU over time was observed for lysates of wt NicA2. For lysate from our best variant in the primary screening assay, A107R, the rate of nicotine degradation was substantially faster than wt. To ensure the data would replicate using fully purified protein and in serum, we compared wt and the improved variant’s capacity to reduce nicotine concentrations in serum using a highly-sensitive and quantitative Gas Chromatographic (GC) assay [32]. The GC assay was only used in this final in vitro assay step since it is low-throughput and would have made it time-consuming to screen all initial hits. As seen in Fig. 2b, the highest activity variant, NicA2A107R, has increased nicotine degrading activity compared to wt NicA2, in serum at nicotine concentrations found in smokers. This enhanced activity replicated and confirmed the biosensor assay results.

With the secondary assays implemented and validated, all variants with at least a 2-fold activity increase in the primary screening assay compared to wt NicA2 were tested at lower nicotine concentrations in the SPR assay, and variants F104 T, I, R & S; G106A; A107R, T, G, F355H and A426C were found to be improved over wt. These hits were subsequently purified and tested in the serum/GC assay. As shown in Fig. 2c, these variants were indeed improved in serum as well.

Simulations of the data in Fig. 2c (Table 2) suggest that at least for one variant (A107R) enhanced *k*_cat_ comes at the expense of increased *K*_m_ (the enzyme concentration needed to attain 50% of the maximum rate of catalysis, *V*_max_). As a validation of the kinetic modeling of low nicotine progress curves, we found wt NicA2 had an apparent *k*_cat_/*K*_m_ of 6.4 × 10^4^ M^− 1^ s^− 1^ in close agreement with prior reports [26]. The variants shown in Fig. 2c showed improved *k*_cat_/*K*_m_ values ranging from 1.7-fold (G106A) to 3–fold higher (A107R) than wt NicA2 (Table 2). Simulation further suggested that compared to wt NicA2 only A107R displayed a significantly increased *K*_m_ (830 nM), a value in close agreement with steady state kinetic analysis performed with this variant across a broad range of nicotine concentrations (data not shown).

**Table 2:** Calculated apparent kinetic parameters of each mutant compared to wt NicA2

Variant	*k*_cat_/*K*_m_ (M^−1^ s^−1^) (apparent)	*k*_cat_ (s^−1^) (apparent)	*K*_m_ (nM) (apparent)
wt NicA2	6.4 × 10^4^	0.015	240
A107R	1.9 × 10^5^	0.159	830
A107T	1.8 × 10^5^	0.050	285
A107G	1.6 × 10^5^	0.053	335
F104 T	1.7 × 10^5^	0.055	330
F104I	1.6 × 10^5^	0.039	240
F104R	1.5 × 10^5^	0.041	270
F104S	1.3 × 10^5^	0.031	250
G106A	1.1 × 10^5^	0.031	280
F355H	1.3 × 10^5^	0.029	220
A426C	1.3 × 10^5^	0.030	230

Apparent *k*_cat_/*K*_m_ values were calculated using the low nicotine assay results (Fig. 2c) using a form of the Michaelis-Menten equation (*v*_0/[E]_ = *k*_cat_/*K*_m_*[S]) and to construct progress curves that were fitted to a simple kinetic model (KinTek) to derive apparent *k*_cat_ values. The resulting *k*_cat_/*K*_m_ and *k*_cat_ values were used to calculate the apparent *K*_m_ values

### Improved in vivo activity of the A107R mutant

**Fig. 3:**
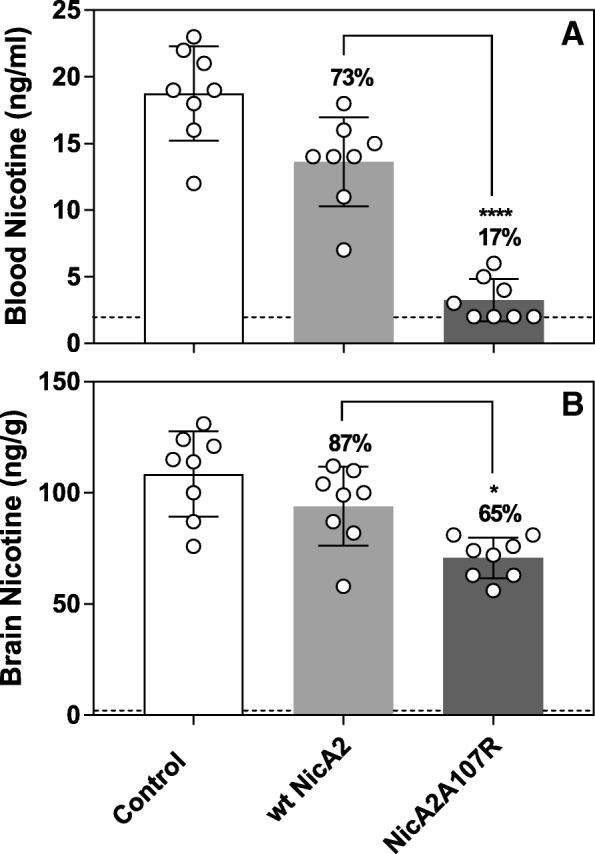
Comparison of the potency of NicA2A107R to wt NicA2 in lowering blood and brain nicotine levels in vivo. Effect of wt NicA2 or NicA2A107R treatment (0.625 mg/kg dosed *i.v.*) on (**a**) blood and (**b**) brain nicotine levels 3 min after 0.03 mg/kg nicotine (i.v; *N* = 8 rats per group). Percent remaining compared to controls are noted above each column. Control group was dosed with BSA. Mean nicotine levels ±SD. Differences between wt NicA2 and NicA2107R were significant: **** *p* < 0.0001, * *p* < 0.05. Dashed line is LOQ: 2 ng/mL for blood, 2 ng/g for brain

In order to ensure our assays would ultimately translate to enhanced in vivo efficacy, the effects of dosing either wt NicA2 or NicA2A107R at 0.625 mg/kg on nicotine distribution to blood and brain were tested. Eight rats per group were pre-dosed with enzyme or bovine serum albumin (BSA; control), and 10 min later given a nicotine dose of 0.03 mg/kg by *i.v.* bolus injection delivered in under 10 s (a nicotine dose equivalent to 2 cigarettes in a human on a mg/kg basis). 3 min later, blood and brains were isolated, enzymatic activity was quenched immediately as previously described [7], and samples analyzed for nicotine content using GC (Fig. 3). NicA2A107R lowered nicotine blood concentrations to 3.25 ± 1.5 ng/mL vs. 13.6 ± 3.1 ng/mL for wt NicA2 (*p* < 0.0001, one-way analysis of variance (ANOVA) with Bonferroni’s correction comparing A107R to wt NicA2) or an 83% vs. 27% reduction, respectively, compared to controls (18.8 ± 3.3 ng/mL). As expected at the 0.625 mg/kg dose, brain nicotine levels were only partially reduced: 35% for NicA2A107R and 13% for wt NicA2 (*p* = 0.02), as compared to a 95% reduction when 20 mg/kg wt NicA2 is dosed [7]. These data suggest that NicA2A107R is approximately 3-fold more potent than wt NicA2 in vivo under these conditions.

### PEGylation increases circulating half-life and reduces the clinical immunogenic potential of NicA2

**Fig. 4:**
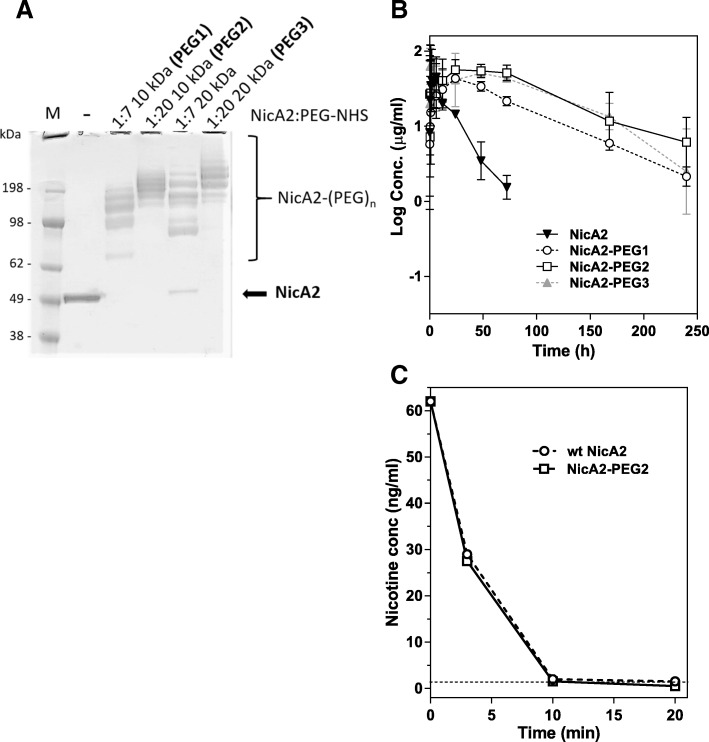
Effects of PEGylation of NicA2. (**a**) SDS-PAGE gel showing mobility of NicA2 (lane 2), conjugated NicA2 using a 1:7 (lane 3) or 1:20 (lane 4) molar excess of a 10 kDa linear or 20 kDa linear (lanes 5 and 6) NHS-PEG reagent. The mobility of unconjugated NicA2 (52 kDa) is indicated by an arrow. Lane 1 contains MW markers. (**b**) Increased circulating half-life of PEGylated NicA2. Wt NicA2 or the 3 different PEGylated preparations were dosed *i.v.* (5 mg/kg) in rats, and concentrations in serum samples retrieved at various time-points (measured by ELISA) plotted as a function of time. (**c**) Identical amounts of purified wt NicA2 (circles) or NicA2-PEG2 (squares) were added to rat serum containing 50 ng/mL S-(−)-nicotine. Samples were withdrawn, and activity quenched by MeOH addition at the time points indicated. Nicotine concentration was measured by GC and plotted as a function of time

Random PEGylation of wt NicA2 was tested using primary amine-reactive PEG-NHS reagents with linear PEG chains of either 10 or 20 kDa (see Methods for details). SDS-PAGE analysis (Fig. 4a) indicated that the degree of PEGylation increases as the molar excess of PEGylation reagent and PEG chain length increases. Preparations of NicA2-PEG1, -PEG2, and -PEG3 where no residual unconjugated protein was detected by SDS-PAGE were chosen for further analysis. In order to determine whether PEGylation could enhance the PK properties of NicA2, serum concentrations were determined as a function of time after *i.v.* dosing in rats (5 mg/kg; *N* = 4). As seen in Fig. 4b, PEGylation significantly enhanced the terminal half-life of NicA2, extending the elimination-phase half-life from approximately 7 to 60 hours (h). No major difference between the 3 PEGylated forms were observed. The potential impact on enzyme activity by PEGylation was tested in the most application-relevant assay, the serum GC assay mentioned above. As seen in Fig. 4c, even the extensive PEGylation of the NicA2-PEG2 preparation did not appear to impede NicA2’s ability to degrade nicotine.

**Fig. 5:**
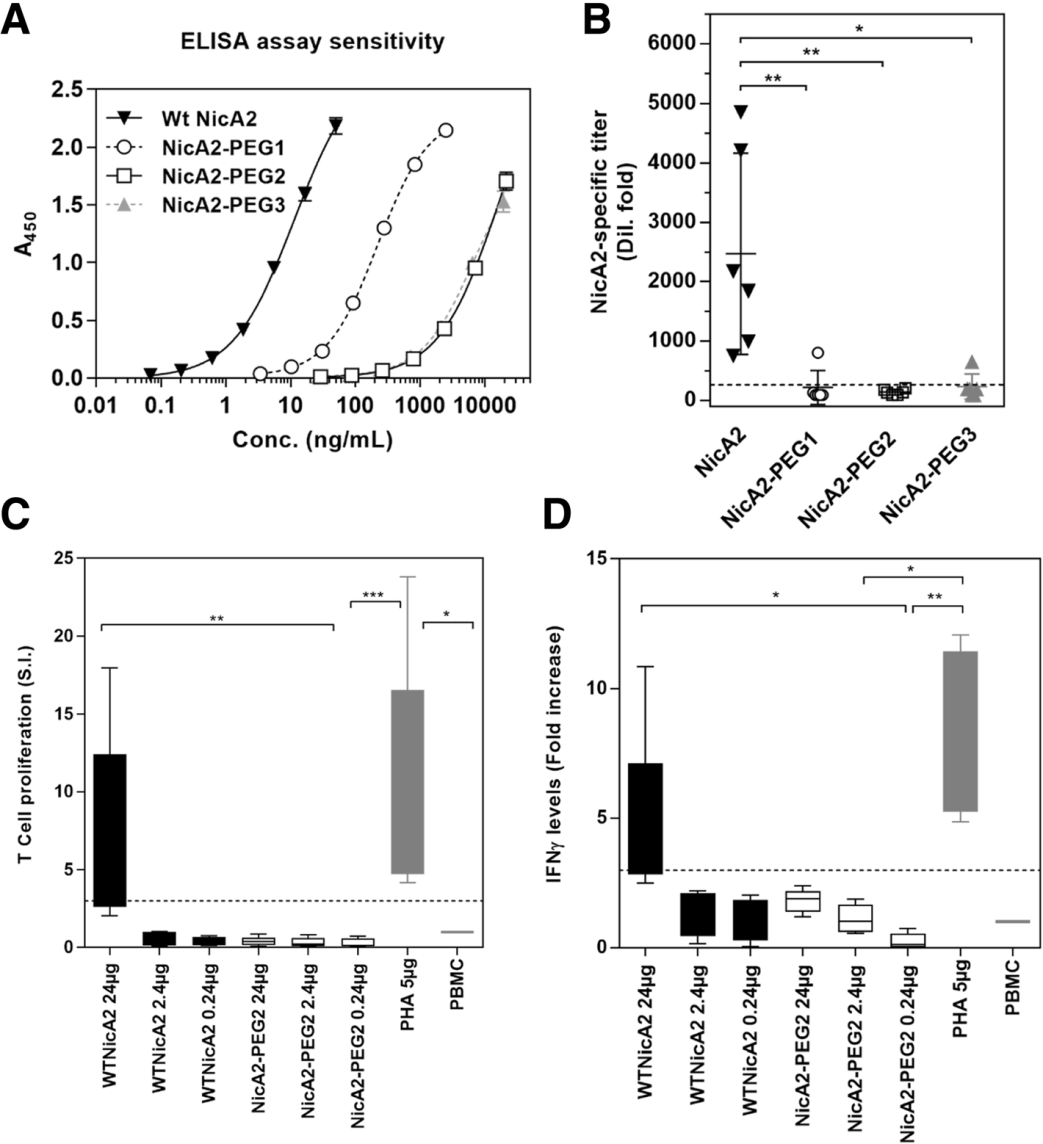
Comparison of PEGylated NicA2 (NicA2-PEG2) to wt NicA2 in assays related to immunogenicity and lymphocyte stimulation. (**a**) Detection of unconjugated NicA2 or various preparations of PEGylated NicA2 in a sandwich ELISA format. (**b**) Immunogenicity in a transgenic mouse model (HLA DR4), * = *p* < 0.01 and ** = *p* < 0.001 compared to NicA2 without PEGylation; and human lymphocyte activation in vitro in the form of T cell proliferation (**c**) and IFN-γ secretion (**d**), * = *p* < 0.01, ** = *p* < 0.001 and *** = *p* < 0.0001 for the comparisons indicated (see Experimental Procedures for details). An increase of ≥3 (dashed line) above the baseline levels is considered a significant increase and a positive response

Serial dilutions in PBS of unPEGylated or PEGylated preparations of NicA2 were tested in the same sandwich ELISA assay used for measurement of serum concentrations in the PK experiment, and signal (A_450_) plotted as a function of concentration. Sensitivity of the assay was dramatically reduced with increasing degree of PEGylation (approx. 1000-fold higher concentration of NicA2-PEG2 and -PEG3 required to obtain an A_450_ of 1.0 relative to unPEGylated enzyme; Fig. 5a), indicating that epitopes recognized by the detection antibody reagents (anti-His tag mAb and rabbit polyclonal antiserum to NicA2) were less accessible in the PEGylated molecules.

PEGylation of NicA2 led to a significant ≥10-fold decrease in average NicA2-specific antibody titers in transgenic HLA-DR4 mice (Fig. 5b; 4, 2, and 2, animals from groups NicA2-PEG1, -PEG2, and -PEG3, respectively, had titers below the LOQ; and *p* = 0.002, *p* = 0.009, *p* = 0.04 using Kruskal-Wallis test and Dunn’s test for multiple comparisons when comparing to NicA2 without PEGylation), indicating that PEGylated variants may exhibit lower immunogenicity in a clinical setting.

In an in vitro assay using cultured human lymphocytes, PEGylation of NicA2 led to a significant decrease in the average human T cell proliferation Stimulation Index (SI; Fig. 5c) as well as decreases in IFN-γ secretion levels (Fig. 5d). Test results for five independent experiments on peripheral blood mononuclear cells (PBMCs) isolated from five healthy volunteers are shown for T cell proliferation response SI and fold-increase in IFN-γ secretion levels (for NicA2 and NicA2-PEG2 at three test concentrations in comparison to baseline responses (PBMC; positive control: phytohaemagglutinin (PHA)) [33, 34]. An increase of ≥3 (dashed line) above the baseline levels is considered a significant increase and a positive response. T cell proliferation responses and IFNγ levels in NicA2-PEG2 samples were significantly lower in comparison to the positive control (PHA) and to the highest concentration of NicA2 indicating low/no immune activation.

### Pilot chronic toxicology testing indicates that NicA2 in the presence of nicotine is well-tolerated

To assess the safety of NicA2 in the presence of nicotine, we conducted a 28 d repeat-dose toxicology study in rats dosing 20 mg/kg of NicA2-ABD (a long-acting form of NicA2 [7, 11]) by *i.v.* injection every 4th day plus nicotine at 1 mg/kg/d via continuous infusion pump (equivalent to > 30 cigarettes worth of nicotine/d on a mg/kg basis). Two control groups, saline or nicotine alone were included. The long-acting NicA2-ABD made this study possible by significantly reducing the dosing frequency and amount of material needed. NicA2-ABD has a circulatory half-life of 61 h [7]. Serum levels of NicA2-ABD at the end of the study averaged 81 μg/mL. NicA2-ABD was well-tolerated with no observed pathology in the treatment group, including assessments of hematology, serum clinical chemistry and histopathologic examination of liver, spleen, heart, lung, kidney, brain, skeletal muscle, stomach, colon, and ovary or testis.

## Discussion

Nicotine is the principal addictive component of tobacco [35] and attenuating its reinforcing effects may help smokers quit and remain quit. An optimized nicotine-degrading enzyme is intended to enhance nicotine elimination sufficiently so that addictive, reinforcing levels of nicotine do not reach the brain of the treated smoker even if they may continue to smoke (i.e. during a quit attempt or upon a lapse), leading to reduced smoking levels, improved rates of cessation, and long-term abstinence. As compared to the current standard of care (varenicline and nicotine replacement therapies), which activate neuronal nicotinic acetylcholine receptors (nAChRs), the enzyme’s elimination of nicotine would stop or attenuate nAChR activation (an antagonistic effect) and reinforcement of the rewarding behavior (smoking), potentially disrupting the cycle of addiction.

Nicotine addiction is recognized as a chronic relapsing disorder, where additional important factors also play a role (e.g. sensory aspects of smoking, situational triggers, etc.) and repeated and sustained efforts at quitting, along with trying different approaches, is often necessary [36]. Therefore, we envision that an optimized nicotine-degrading enzyme will provide a new tool for physicians to utilize in an integrated treatment and relapse prevention plan that includes behavioral counseling and may also incorporate other pharmacotherapies to help address cravings and withdrawals, if needed (e.g. co-administration with varenicline or bupropion, which are not enzyme substrates).

We initiated optimization of the bacterial nicotine-degrading enzyme, NicA2, in order to engineer increased catalytic activity and reduced immunogenic potential improving its suitability for human therapeutic use. Improved catalytic activity will allow a lower total dose of protein, more convenient routes and reduced frequency of administration.

We found it to be relatively straightforward to identify variants with improved catalytic rates (*k*_cat_) at higher concentrations of nicotine; but only a subset displayed improved rates of nicotine turnover at the low substrate (nicotine) concentrations seen in smokers. Simulations suggested that overall for the best variants shown in Fig. 2c, the most important kinetic parameter explaining the activity of the variants is the apparent 2nd order rate constant (*k*_cat_/*K*_m_) that allows these variants to outperform the wt enzyme at low nicotine concentrations (Table 2).

**Fig. 6:**
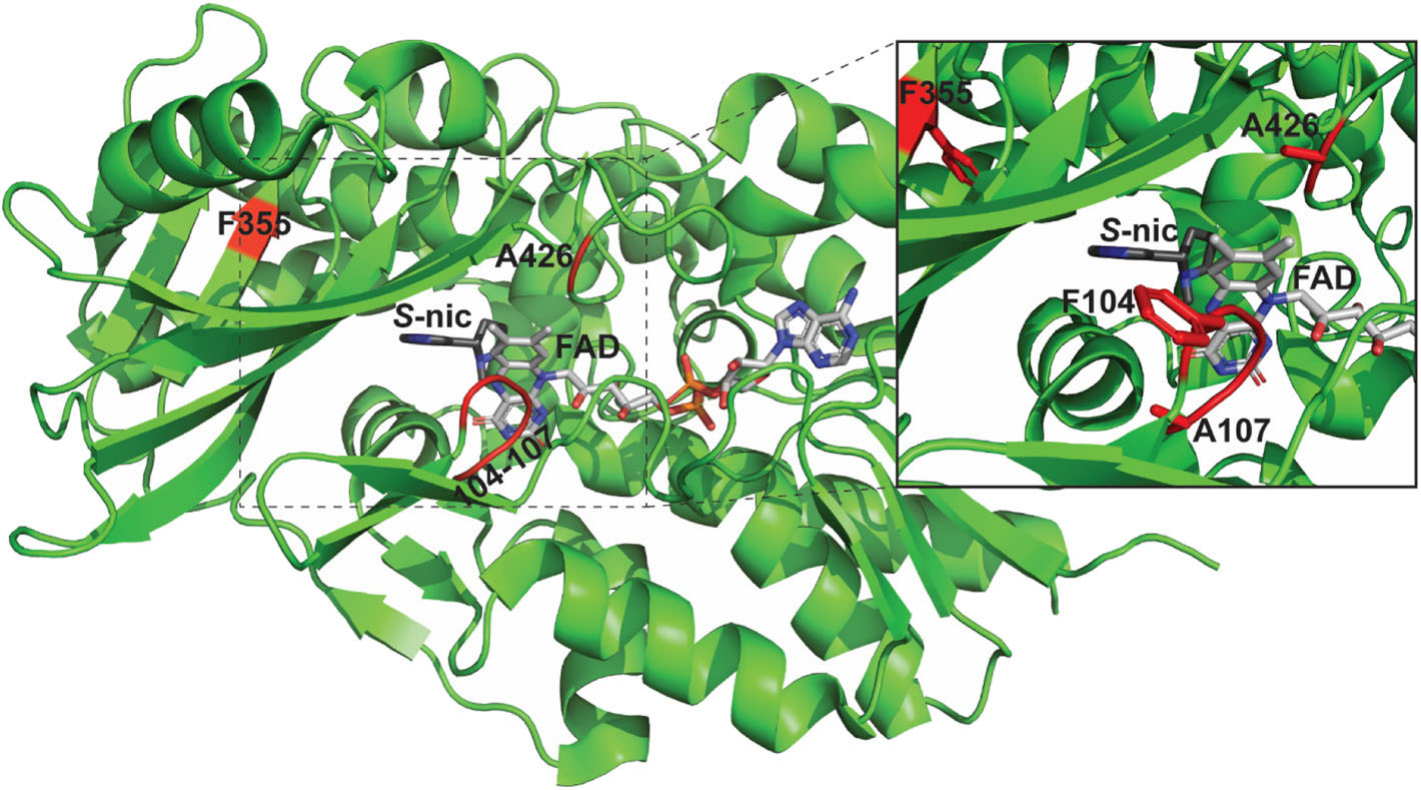
Structure of NicA2/nicotine complex. The 5 mutated residues F104, G106, A107, F355 & A426 that yielded improved nicotine degrading activity are highlighted in red. Modeled from PDB:6C71

Intriguingly, 8 out of 10 variants showing significantly improved activity all contain mutations within a small region in the active site (Fig. 6, residues 104–107). The X-ray crystal structure reveals that nicotine binds in the active site at the *re* face of the flavin ring [26, 27] (Fig. 6); residues F104, G106 and A107 form the majority of a loop on the *si* face of the flavin cofactor near structural tunnels presumed to allow for O_2_ to enter the active site during the second step of the catalytic reaction [27]. As the rate limiting step has been posited to be re-oxidation of the reduced flavin adenine dinucleotide (FAD) [27], mutations at F104, G106 and A107 are well-positioned to facilitate enhanced O_2_ access. Also these mutated positions are within 4–6 Å of K340 which is conserved in the monoamine oxidase (MAO) superfamily and shown to play a critical role in oxidation of FAD in homologous enzymes [37]. As the improved variants all display increased apparent *k*_cat_ values, our hypothesis is that mutations opening-up access to the active site allow for enhanced re-oxidation of the flavin co-factor by O_2_, leading to an overall increased turnover rate. Along these lines we note that the A107R variant with the largest substitution, also displays the largest increase in apparent *k*_cat_ (> 10-fold). As H_2_O_2_ formation rates are greatly enhanced in these variants, which occurs during re-oxidation of the reduced FAD, it suggests that A107R/T/G, F104 T/I/R/S and G106A have all increased the rate limiting re-oxidation step by increasing O_2_ access or by positioning K340 more favorably within the active site.

The A426C substitution (Fig. 6) is particularly interesting as in the structurally homologous MAOs this position contains a conserved Cys residue that is covalently bound to the flavin ring [38]. This residue is adjacent to W427 which forms part of the nicotine binding pocket and close to the *re* face near the methyl groups of FAD (Fig. 6). Although beyond the scope of this manuscript we speculate that the A426C substitution may either covalently constrain or reposition the flavin ring closer to the side chain of K340 during the re-oxidation step, as the A426C variant shows a two-fold increase in apparent *k*_cat_ (Table 2). The rationale behind the improvement observed in the last mutation, F355H, is not immediately obvious, and further analysis (including X-ray crystallography) is needed to elucidate its effect.

All together our data suggest that NicA2A107R is approximately 3-fold more potent than wt NicA2 in vivo and establishes the crucial correlation between improved activity in our in vitro screening assays and improved in vivo potency.

In considering further development of an optimized nicotine-degrading enzyme for potential human therapeutic use, understanding if chronic administration leads to gross toxicities arising from the enzyme’s metabolites or due to the enzyme itself (e.g. degrading an endogenous substrate needed for normal physiological functioning) is critically important. Pseudooxynicotine (PON) is the primary product of NicA2-catalyzed nicotine metabolism and is further metabolized into a non-toxic keto acid. PON is also generated in smokers via 2′-oxidation of nicotine by CYP2A6 [10], and does not appear to have any significant toxicities. In addition, hydrogen peroxide (H_2_O_2_) is generated when the FAD co-factor is re-oxidized by O_2_ after nicotine’s oxidation. H_2_O_2_ can generate reactive oxygen species causing damage to cellular proteins and processes and could pose a potential toxicity risk. Results from a repeat-dose toxicology study which dosed 20 mg/kg every 4 d for 28 d (or a total of 140 mg/kg) in the presence of continuously infused nicotine indicate that NicA2 and its metabolites are not toxic and are well-tolerated as no treatment related pathologies were observed in this pilot study at this dose level. An important future study will be to test the final optimized enzyme candidate selected for development at a range of higher doses to further evaluate its toxicological profile.

While NicA2 is of bacterial origin and carries risk of clinical immunogenicity, many engineered proteins entirely of non-human origin or animal-human chimera have been approved by regulatory authorities for human use [39]. PEGylation is a frequently used option to reduce immunogenicity. An example is Krystexxa for chronic administration in gout patients. Even though PEGylation attenuates the immune response to this tetrameric pig-baboon chimera, anti-drug and anti-PEG response is observed in a substantial fraction of patients upon chronic dosing [40]. In the case of a potential NicA2-based therapeutic for smoking cessation, the duration of treatment is expected to be relatively brief, comparable to the 12-week treatment period of current smoking cessation drugs. This relatively short expected duration of treatment should reduce the opportunity for development of neutralizing anti-drug antibodies (ADAs, either anti-NicA2 or anti-PEG antibodies).

While no highly predictive animal models of human immunogenicity exist, our studies of NicA2 indicate that PEGylation is an effective approach to lower immunogenicity in a transgenic mouse model carrying a human MHC class II allele. Moreover, this was further corroborated using human in vitro testing, as lower activation and proliferation of human lymphocytes in response to NicA2 exposure was observed. Interferon gamma plays an important role in immune response and can be used as a measure of immune activation. IFN-γ secretion in lymphocyte cell culture supernatants in response to NicA2 exposure was reduced, further supporting the lowering of immunogenic potential of NicA2 following PEGylation. In addition, PEGylation significantly extended the enzyme’s serum half-life without affecting catalytic activity.

Next steps towards clinical application are screening residues outside the active site and combinations of improved variants to determine if further increases in activity can be found to achieve reductions in effective enzyme dosages. Ultimately, a PEGylated candidate with sufficiently enhanced nicotine-degrading activity could allow convenient dosing and provide a viable therapeutic development candidate.

Limitations of this research are that only residues at or near the active site residues were mutated and changes within the remaining 461 residues may also lead to improvements in catalytic activity. In addition, this work is limited to reporting single point mutations and combinations of the identified improved variants is an important continuing effort. Kinetic simulations were conducted on a limited number of samplings. The enzyme dose used in the pilot toxicology study only evaluated a dose approximating the therapeutically effective dose of the wt enzyme. Toxicology studies with much higher doses of the final optimized development candidate will be needed to determine the no observed adverse effects level and dose ranges that are likely to be safe for human use.

## Conclusions

Systematic mutational analysis of the active site of the nicotine-degrading enzyme NicA2 has yielded 10 variants that increase its catalytic activity and its effects on nicotine distribution in vivo at nicotine plasma concentrations found in smokers. One of the most improved variants A107R is approximately three-fold more potent than wt at reducing nicotine distribution to brain in rats. In addition, PEGylation of the enzyme substantially increases its circulating half-life and reduces the enzyme’s immunogenic potential. Taken together, these improvements provide a viable path towards generation of a drug candidate suitable for human therapeutic use in treating nicotine addiction with the promise of smaller, less frequent doses, and a reduced potential for immunogenicity.

## Methods

### 3D molecular graphical visualization

The NicA2 protein was visualized using Discovery Studio 4.5 (Dassault Systemes, BIOVIA Corp., San Diego CA) to determine active site residues. Based on inspection of the structure (PDB ID# 5TTJ), location of FAD and reporting of putative critical active site residues [26], a presumed active site cavity was defined. All residues making up the exposed surface of this cavity, including both side chains and backbone atoms, were classified as active site residues: R9, F104, G105, G106, A107, W108, L217, Y214, Y218, R249, T250, K340, P355, W364, Q366, T381, W17, A426, W427, A61, N462, I463.

### Expression and purification of NicA2

Cloning, expression and purification of C-terminal His_6_-tagged NicA2 was done as previously described [7].

### Activity assay on purified proteins

Analysis of enzymatic activity on purified protein was conducted using an Amplex Red assay [28] as previously described [7]. Briefly, the oxidation of nicotine by NicA2 results in generation of H_2_O_2_ which is coupled to the conversion of the colorless Amplex Red reagent into its red-fluorescent product, resorufin by HRP (horseradish peroxidase), Fig. 1a. The assay was performed essentially as recommended by the supplier of the assay kit (Thermo; Cat# A22188) with the exception of addition of S-(−)-nicotine (Sigma) to a final assay concentration of 10 μM. Activities were expressed as the relative slopes of increase in fluorescence plotted as a function of time compared to the wt NicA2 enzyme, which was run in parallel.

### Saturation mutagenesis library generation

Eight single position libraries (positions R91, L217, T250, K340, Q366, T381, N462, I463) were generated by QuikChange Site-Directed Mutagenesis (Agilent) using DNA oligonucleotides carrying an NNK randomization of the relevant codons (Integrated DNA Technologies) and a pET22 expression vector encoding NicA2Δ50 as template. The resulting libraries each had an NNK randomization of a unique codon resulting in a 32-member library (at the DNA/codon level) encoding variants with all possible 20 amino acids in said position. The libraries were transformed into the expression strain BL21 Gold(DE3) (Agilent). A total of 176 random colonies per library were screened to ensure sufficient coverage (theoretical 3-fold oversampling of each library member).

To cover the remaining active site positions, a custom CSM library was supplied by Revolve Biotechnologies, Inc. [30], probing all single amino acid substitutions (one mutation per variant) in the following positions of full-length NicA2: F104, G105, G106, A107, W108, Y214, Y218, E249, F355, W364, W417, A426, and A461. Of 24 random clones sequenced for library QC, one clone was wt (encoded by a different codon), 22 were single point mutations in 8 out of the 13 designed positions, whereas one clone carried an additional fortuitous point mutation in an unrelated position. A total of 12 plates (1056 random clones) were screened from this library of theoretical 416 members (codon level), i.e. 2.5-fold oversampling of each library member assuming ideal library diversity.

### Primary enzyme screening assay

Single colonies were picked and grown overnight in a 96 well plate in LB media containing Carbenicillin (100 μg/mL). Overnight LB cultures were diluted into a new 96-deep well plate containing 475 μl of auto-inducing Magic Media (Invitrogen) + Carbenicillin (100 μg/mL) and grown for 18 h at room temperature with vigorous shaking. Bacteria were harvested in the plate by centrifugation at 4000 rpm for 15 min, and pellets frozen at − 80 °C. Pellets were lysed by dissolving in 100 μl of room temperature Bug Buster HT Reagent (Novagen) containing 1KU of r-Lysozyme per 1 mL and incubating on a shaking platform for 20 min at room temperature. Cleared lysates were prepared by diluting 1:1 (vol.:vol.) with Bug Buster HT Reagent and removing insoluble cell debris by centrifugation at 4400 rpm for 20 min at 4 °C. 25 μl of cleared lysates were transferred to a new 96 well plate and diluted 1:1 in 2% milk in PBS. Diluted lysates were transferred to the assay plate (black 96-well half area high binding plate (Corning) coated o/n at 4 °C with 5 μg/mL of anti-His Tag antibody (R&D Systems) in PBS; 50 μl per well; then blocked with 4% milk (in PBS) for 2 h at RT) and incubated at room temperature gently shaking for 3 h to ensure saturation of the immobilized anti-His mAb with the molar excess of expressed His-tagged enzyme. A further 25-fold dilution of wt lysate did not result in a decrease in assay signal, indicating this step essentially results in normalizing any differences in concentration afforded by differences in growth, induction conditions, intrinsic expression levels, etc., and ensures a consistent amount of enzyme is assayed for activity in each well in the subsequent steps. This also ablates the need for quantification of enzyme in individual wells to precisely measure and compare activity of variants. Plate was washed 6x with PBST and 1x with Amplex Red Reagent Buffer (Thermo) to remove unbound material. Enzyme was assayed by adding 50 μl of Amplex Red Solution including 10 μM S-(−)-nicotine to each well and monitoring development of fluorescence over time in a SpectraMax M2 plate reader (Molecular Devices) using the settings Ex at 555 nm; Em at 590 nm, and employing a “Plate Blank” well (without added lysate) to subtract background fluorescence.

From each 96 well assay plate, the eleven (11) random colonies expressing variants with the highest activity at least 2-fold above wt (compared to values from 4 included independent colonies expressing wt NicA2 on each assay plate) were re-streaked from the original master plate with overnight LB culture onto agar plates and 8 individual colonies rescreened in the same assay. Plasmid DNA was prepared and sequenced.

#### Variability of the primary screening assay

Assays were conducted on lysates from either random individually picked colonies of *E. coli* expressing NicA2, or a single lysate prepared from one 5 mL culture and subsequently aliquoted out into separate assay wells. Very little sample variation was observed: For the 36 individually grown cultures, average activity (expressed as the slope of relative fluorescence units (RFU) vs. time) was 6480 with a standard deviation of 562, and for the 46 assay wells prepared using the same lysate the average was 7531 with a standard deviation of 331. Based on these observations, detection of a 2-fold improvement (i.e. > 12,000) over wt (6480 ± 562, 95% CI = 203) would be considered significant and constitute a reasonable cut-off for hit identification in the primary screening assay.

### Secondary enzyme screening assays

#### Nicotine biosensor assay

The experiments were conducted using a BiOptix 404pi enhanced SPR instrument. Nicotine-specific mAb, ATI-1119, an IgG1 with a suitable *K*_D_ in the middle of the concentration range to be measured (66 nM for S-(−)-nicotine) was immobilized on a BiOptix CMD200m sensor chip using standard EDC/NHS amine coupling with blocking of the remaining active esters with ethanolamine. For standard curves, S-(−)-nicotine was injected at different concentrations (3x serial dilutions) in running buffer (10 mM HEPES (pH 7.4), 150 mM NaCl, 3 mM EDTA, 0.05% Tween-20). Each injection was followed by buffer flow at 25 °C for 20 min to dissociate nicotine from the mAb and prepare the chip for the next sample injection. Three independent randomized injections were conducted for each nicotine concentration. RUs at steady state was determined using the Scrubber2 software.

For the assay of enzyme activity, crude lysates were prepared as described for the primary screening assay. Enzyme was captured from lysates using immobilized anti-His tag mAb as described above (one well per assay point). After washing off unbound material, the assay was started by adding 100 μl buffer (same as running buffer) with a starting nicotine concentration of 250 nM to mimic the blood concentration encountered in a typical smoker (40 ng/mL) [31]. Reactions were stopped by transferring 90 μl into prelabelled PCR-tubes and heated to 90 °C/10 min to inactivate potential residual enzyme activity. Samples were transferred to a 96 well sample plate, which was subsequently loaded into the autosampler (kept at 10 °C) of the BiOptix instrument.

#### Serum activity assay

Purified enzyme was added to a final concentration of 1.5 μg/mL into 500 μl rat serum containing 40 ng/mL of S-(−)-nicotine (250 nM) pre-incubated at 37 °C. Enzymatic activity was immediately quenched after a given incubation time by addition of 1 mL methanol and rapid mixing, followed by sample storage at − 80 °C [7]. Residual nicotine concentrations were determined by GC with nitrogen phosphorous detection [41, 42]. LOQ of the GC assay is 2 ng/mL for blood and 2 ng/g for brain.

### Kinetic fitting and simulation

To assess the apparent kinetic parameters of NicA2 and derived variants, the data from the low nicotine assay experiments were used to both directly calculate apparent *k*_cat_/*K*_m_ values using a form of the Michaelis-Menten equation (*v*_0/[E]_ = *k*_cat_/*K*_m_*[S]) and to construct progress curves. The progress curves were fit to a simple kinetic model using KinTek software (KinTek Corporation, Snow Shoe, PA) using methodology as described to derive apparent *k*_cat_ values [43]. The resulting *k*_cat_/*K*_m_ and *k*_cat_ values were used to derive the apparent *K*_m_ values.

### Rat nicotine distribution studies

Male and female Sprague Dawley rats weighing approximately 300 and 250 g, respectively, were purchased with jugular venous catheters in-place (Charles River Labs). Three groups of 8 rats (4 males + 4 females per group) were pretreated with BSA, wt NicA2, or NicA2A107R through the catheter at a dose of 0.625 mg/kg. A minimum of 5 min later each group received 0.03 mg/kg nicotine *i.v.*. Rats were sacrificed at 3 min following the nicotine dose. Blood and brain samples were obtained by decapitation and quenched with methanol as previously described [7]. Blood or brain nicotine concentrations were compared by one-way ANOVA with a Bonferroni correction for multiple comparisons. These studies were conducted by Noble Life Sciences.

### Toxicology testing of NicA2 in the presence of nicotine

The purpose of this study was to evaluate the repeat-dose tolerability of seven (7) fixed *i.v.* doses of 20 mg/kg NicA2-ABD dosed once every 4 d (140 mg/kg in total) in the presence of 1 mg/kg/d of nicotine given continuously by *i.v.* for 28 d using a rat model. This dose amount was selected since 20 mg/kg was sufficient to reduce brain nicotine levels by 95% in a rat nicotine-PK study [7] and drug supply was limited.

Twenty (20) female and twenty (20) male Sprague Dawley rats (225–300 g) were divided into two groups of sixteen (16) animals and one group of eight (8) animals. Animals were implanted with osmotic pumps delivering a continuous dose of nicotine or vehicle through the study period. NicA2-ABD (every 4 d) was delivered by intravenous injection. 16 animals received saline, 16 animals received nicotine only and only 8 animals received NicA2-ABD due to limited available drug supply.

All animals received the full dose (7 × 20 mg/kg *i.v.*) with no injection related behavioral changes, injection site reaction, and no mortality was induced in any of the animals. Daily clinical observations found no observable behavioral changes or modifications in feeding or grooming in any groups. Body weight was monitored twice weekly for the duration of the study and no significant differences between treatment groups were found. On Study day 28, blood was collected and analyzed for hematology, serum chemistry, and coagulation. Additional aliquots of blood plasma were taken for assaying blood levels of NicA2-ABD. After blood collection animals were placed under anesthesia with isoflurane and sacrificed by thoracotomy.

Assessment of toxicity was based on mortality, clinical observations, and body weight during the 28 d study; and at the end of study on organ weights, gross anatomic pathology, hematology, serum clinical chemistry, and coagulation.

At the end of the study animals were necropsied with no gross pathological finding noted in any animal. Major organs (liver, lung, spleen, heart, kidneys, testis or ovaries) were isolated and weighed. No gross pathological findings were noted in these tissues, and no statistically significant changes in organ weights were found (kidneys, testis, and ovaries weighted as a pair). Blood was collected, and complete blood count performed to determine any changes in hematological parameters. While occasional animals had values outside the normal range (e.g. slightly decreased lymphocytes or hemoglobin) no significant changes or trends were found in any group. There was a trend to have slight polychromasia in some of the control animals that received nicotine alone. Serum clinical chemistry of 23 different analytes and plasma coagulation measures did not find any notable changes between treatment groups.

Tissue histopathology evaluations for heart, liver, lung, kidney, spleen, skeletal muscle, brain, colon, stomach, ovary, and testis have been conducted. Tissues were fixed immediately in formalin, embedding in paraffin, staining with H&E, and were reviewed by a veterinary pathologist. Histopathological examination found no test article related lesions in any tissue examined. Tissues were specifically examined for any evidence of an immune histopathologic reaction and none were observed at the 20 mg/mL dose tested. These studies were conducted by Noble Life Sciences.

### PEGylation of NicA2

Random PEGylation of wt NicA2 was performed at a protein concentration of 5 mg/mL and using 10 or 20 kDa NHS-PEG reagent (Sunbright® ME-100TS or ME-200TS; NOF America Corporation) at 7- or 20-fold molar excess in 100 mM Na_3_PO_4_, pH 7.6 on ice for ≥2 h. Elimination of unconjugated PEG reagent was performed using Amicon Ultra-15 centrifugal filter units with a 50 kDa cutoff. Samples corresponding to 2 μg protein were analyzed on SDS-PAGE gels run in MOPS running buffer and stained using SimplyBlue SafeStain (Invitrogen).

### Serum elimination rate of PEGylated NicA2

Sprague Dawley rats were obtained with a jugular venous catheter in place (Charles River). Four groups of 4 rats (2 male and 2 female) received 5 mg/kg (protein concentration) His-tagged NicA2, NicA2-PEG1, −PEG2, or -PEG3 by intravenous injection via the lateral tail vein. Blood (0.2 mL) was collected into serum separator tubes via the jugular catheter at pre-dose and over a 5 min-72 h period for NicA2 or 5 min-10 d for NicA2-PEG1, -PEG2, and -PEG3, and serum was isolated and stored at −20 °C until analysis. Assay of NicA2 concentrations in serum samples was performed by ELISA as previously described [7]. Briefly, ELISA plates coated with anti-His tag antibody (R&D Systems), was used to capture NicA2 and PEGylated NicA2 proteins through the C-terminal His-tag, and detection performed with a rabbit anti-NicA2 polyclonal antibody (Noble Life Sciences) and horseradish peroxidase (HRP)-conjugated goat anti-rabbit IgG (Fc) (KPL International). Estimates of pharmacokinetic parameters (volume of distribution, clearance, terminal half-life) were calculated using noncompartmental methods [44]. These studies were conducted by Noble Life Sciences.

### Assessment of immunogenic potential in a human HLA-DR4 mouse model

The reduction of NicA2-specific antibody titers 10 d after subcutaneous (*s.c.*) injection in Freunds Incomplete Adjuvant in human DR4 transgenic mice (*N* = 6; 3 M + 3F; Taconic Biosciences) was studied. This mouse model carries a hybrid MHC class II molecule with the antigen binding domains of human HLA-DRA and HLA-DRB*0401 (representative of the DR4 supertype) and does not express endogenous mouse MHC class II molecules [45]. Titer was defined as serum dilution to achieve OD450 = 0.5 in ELISA using NicA2 coated plates, and detection by goat α-mouse IgG-ɣ-HRP. The lowest serum dilution tested was 50-fold (Limit of Detection (LOD)). Titers from NicA2-PEG1, −PEG2, and PEG3 were compared to unPEGylated NicA2 using one-way ANOVA using Kruskal-Wallis test and Dunn’s test for multiple comparison. These studies were conducted by Noble Life Sciences.

### T cell stimulation and IFN-γ secretion assays in human T cells

Local Research Ethics Committee (LREC, Northeast Newcastle, UK) approval was granted prior to study commencement. Peripheral blood (60 mL) was obtained from healthy volunteers (*n* = 5) after receiving their written informed consent. PBMCs were isolated using density-gradient centrifugation (Lymphoprep™, Stemcell Technologies) and then used for positive selection of CD14^+^ monocytes. The CD14^−^ fraction was collected and used as a source of autologous lymphocytes. Monocyte-derived dendritic cells (MoDC) were generated as previously described [46]. T cell proliferation assays were performed by incubating the test compounds with autologous MoDC and then activation by autologous T cells for 5 d. Each sample was set up in triplet wells. Both wt NicA2 and NicA2-PEG2 were tested at 24 μg/mL, 2.4 μg/mL and 0.24 μg/mL. PHA, 5 μg/mL) was used as a positive control. To determine baseline proliferation untreated MoDCs were co-cultured with autologous lymphocytes. Supernatants were collected for IFN-γ analysis prior to [^3^H] thymidine addition on day 5.

T cell proliferation was measured by [^3^H]-Thymidine incorporation in counts per minute (cpm). Analysis was performed by calculating a stimulation index of T cell proliferation by dividing the cpm value obtained for test samples with the baseline cpm value. A fold increase in IFN-γ levels (pg/mL) (measured by flow cytometry using a Cytometric Bead Array kit, BD Biosciences) was calculated by dividing the value of cells treated with each drug with the baseline value. The cut off value of a 3-fold increase [47] was considered to be a positive response. Statistical analysis was performed using one-way ANOVA using Kruskal-Wallis test and Dunn’s test for multiple comparison. These data were generated by Alcyomics Ltd., UK.

### Animal husbandry

Equal numbers of age-matched male and female Sprague Dawley rats (Charles River Laboratories) or HLA transgenic mice per study group were used. Study animals with surgical modifications were housed individually in disposable microisolator cages (Innovive). Environmental controls were set to maintain the following conditions: a temperature range of 64 to 79 °F, a relative humidity range of 30 to 70%, ten or greater air changes/h, and a 12-h light/12-h dark cycle. Food and water were available ad libitum. Animal welfare followed the NIH guide for Care and Use of Laboratory Animals (8th ed.) and all protocols were approved by Noble Life Sciences (NLS) Institutional Animal Care and Use Committee. NLS is an AAALACi accredited and USDA Licensed (51-12-0092) and OLAW Assured (A4633–01) facility. Collection of blood from animals occurred while under isoflurane anesthesia and steps necessary to minimize animal suffering were undertaken. Study animals were euthanized after terminal blood collection by thoracotomy under isoflurane anesthesia consistent with AVMA Guidelines.

## Data Availability

Data associated with this study are available from the corresponding author upon reasonable request.

## Abbreviations

ABD: Albumin binding domain
CSM: Comprehensive Saturation Mutagenesis
FAD: Flavin adenine dinucleotide
NAD: Nicotinamide adenine dinucleotide
PEG: Polyethylene glycol
PON: Pseudooxynicotine
wt: Wild-type
